# Impact of Fluorescent In Situ Hybridization Aberrations and CLLU1 Expression on the Prognosis of Chronic Lymphocytic Leukemia: Presentation of 156 Patients from Turkey

**DOI:** 10.4274/tjh.2017.0112

**Published:** 2018-03-06

**Authors:** Ümmet Abur, Gönül Oğur, Ömer Salih Akar, Engin Altundağ, Huri Sema Aymelek, Düzgün Özatlı, Mehmet Turgut

**Affiliations:** 1 Ondokuz Mayıs University Faculty of Medicine, Department of Medical Genetics, Samsun, Turkey; 2 Ondokuz Mayıs University Faculty of Medicine, Department of Hematology, Samsun, Turkey

**Keywords:** Chronic leukemia, Chronic lymphocytic leukemia, Cytogenetics/FISH, CLLU1

## Abstract

**Objective:**

This study evaluates the impact of CLLU1 expression and fluorescent in situ hybridization (FISH) analysis of a group of Turkish chronic lymphocytic leukemia (CLL) patients.

**Materials and Methods:**

A total of 156 CLL patients were analyzed by FISH method; 47 of them were also evaluated for CLLU1 expression. Results were correlated with clinical parameters.

**Results:**

FISH aberrations were found in 62% of patients. These aberrations were del13q14 (67%), trisomy 12 (27%), del11q22 (19%), del17p (8%), and 14q32 rearrangements (20%). Overall del11q22 and del17p were associated with the highest mortality rates, shortest overall survival (OS), and highest need for medication. Homozygous del13q14, 14q32 rearrangements, and higher CLLU1 expression correlated with shorter OS.

**Conclusion:**

Cytogenetics/FISH analysis is still indicated for routine evaluation of CLL. Special consideration is needed for the poor prognostic implications of del11q22, del17p, 14q32 rearrangements, and homozygous del13q14. The impact of CLLU1 expression is not yet clear and it requires more data before becoming routine in genetic testing in CLL patients.

## Abstract (tr)

**Amaç:**

Bu çalışma, bir grup Türk kronik lenfositik lösemi (KLL) hastasında CLLU1 ekspresyonu ve floresan in situ hibridizasyon (FISH) analizinin prognostik etkisini değerlendirmektedir.

**Gereç ve Yöntemler:**

Yüz elli altı KLL hastası FISH yöntemiyle analiz edildi. Bu 156 hastanın 47’sinde ek olarak CLLU1 ekspresyonu incelendi. Sonuçlar klinik parametrelerle ilişkilendirildi.

**Bulgular:**

FISH aberasyonu, hastaların %62’sinde bulundu. Aberasyonların dağılımı del13q14 (%67), trizomi 12 (%27), del11q22 (%19), del17p (%8) ve 14q32’nin yeniden düzenlenmesi (%20) olarak bulundu. En yüksek mortalite, en kısa sağkalım süresi ve en fazla ilaç kullanımı del11q22 ve del17p grubunda idi. Homozigot 13q14 delesyonu, 14q32 yeniden düzenlenmesi ve yüksek CLLU1 ekspresyonu olan hastalar kısa sağkalıma sahipti.

**Sonuç:**

Sitogenetik/FISH analizi, KLL’nin prognostik değerlendirmesinde ve yeni genetik moleküler belirteçlerin belirlenmesinde halen etkili yöntemlerdir. del11q22, del17p, 14q32 yeniden düzenlenmesi ve homozigot del13q14’ün kötü prognostik etkisi gözden kaçırılmamalıdır. CLLU1’in KLL’de prognostik yeri tartışmalıdır. Çalışmamızda orta-kötü prognostik bir kriter olarak belirmesine rağmen, KLL’de rutin genetik testler arasına girebilmesi için daha fazla veri gereklidir.

## Introduction

The clinical manifestation of chronic lymphocytic leukemia (CLL) is variable. While some patients are asymptomatic for years, others show a rapid progression of the disease [1]. Recent identifiers of high-risk patients include chromosomal abnormalities, immunoglobulin heavy chain variable gene, ZAP70, CD38, b2 microglobulin and lactate dehydrogenase (LDH), and CLL upregulated gene 1 (*CLLU1*) expression [2]. The chromosomal abnormality rate in CLL is 30%-50%; this rate reaches up to 70%-80% with the fluorescent in situ hybridization (FISH) method [3,4]. FISH results have shown that del13q14 is correlated with good prognosis whereas del11q22 and del17p indicate poor prognosis [5,6].

Unfortunately, CLL is genetically heterogeneous. Recently relevant new genomic abnormalities such as *NOTCH1* and *SF3B1* mutations as well as *BIRC3* disruptions have been described [7,8], but none of these genetic markers are unique to CLL. *CLLU1* is defined as the first gene specific to CLL. The high expression level of *CLLU1* seems to be unique in CLL [9]. However, its relevance to prognosis is still unclear.

In this study, the distribution and prognostic impact of chromosomal aberrations via FISH as well as *CLLU1* expression levels were analyzed in a group of North Anatolian CLL patients. 

## Materials and Methods

### Patients

Interphase FISH analysis was applied to blood or bone marrow samples of 156 CLL patients. Of these, 47 were also evaluated for *CLLU1* expression and compared with 35 healthy controls. Staging was done according to the modified Rai staging (MRS) system. The results of the b2 microglobulin, LDH, white blood cell (WBC) count, and absolute lymphocyte count were grouped as high or low risk (Table 1).

FISH data were categorized as group 1: del13q14, group 2: trisomy 12, group 3: del11q and del17p, and group 4: normal FISH results. Additionally, two groups were formed with 14q32(IGH) rearrangements being positive or normal.

### Interphase FISH

FISH analysis was performed by directly labeled probes (Vysis/Abbott Co., Abbott Park, IL, USA). A FISH panel of 5 probes (D13S319, LSI 13q34, LSI ATM, CEP12, LSI p53) was applied [10]. Seventy-one out of 156 patients were also tested by 14q32 break-apart probe.

FISH analyses were conducted using an Olympus BX51 microscope equipped with a Progressive Scan Video Camera (Tokyo, Japan). Image analysis was carried out with CytoVision software (version 3.93; Applied Imaging, Grand Rapids, MI, USA). For each probe for optimization, a cut-off level was obtained by counting 300 cells. Results were considered clonal when the percentage of cells with any given chromosome abnormality exceeded the normal cut-off value.

### CLLU1 Expression

For the analysis of CLLU1 expression, RNA was isolated (QIAGEN, Hilden, Germany); cDNA was synthesized using a cDNA Reverse Transcription Kit (Ipsogen, QIAGEN). *CLLU1* expression was tested by real time-polymerase chain reaction (Rotor-Gene Q, QIAGEN) using primers/probes previously defined (Ipsogen, *CLLU1* Profile Quant Kit). Analysis was performed using the comparative Ct method of relative quantification with b2 microglobulin as an endogenous control. The *CLLU1* expression levels were measured as fold upregulation in relation to normal patients’ cells and a cut-off value was defined to separate high from low expression levels [11].

### Statistical Analysis

The chi-square test was applied to determine the relationship among clinical and laboratory parameters (LDH and b2 microglobulin, WBC, MRS, *CLLU1* expression, and subsets of FISH abnormalities). Overall survival (OS) was tested by the Kaplan-Meier method. The survival curves were statistically compared using a log-rank test (p≤0.05). 

## Results

### Patient Population

Of 156 patients, 103 patients were male. Ages ranged from 36 to 90 years (median: 68 years). In total, 37 patients died during the study. The median OS time was 101±12 months. 

### Results of FISH

FISH analysis detected aberrations in 96 patients (62%). The most frequent abnormality was del13q14 (67%), followed by trisomy 12 (27%), del11q22 (19%), and del17p13 (8%). The occurrence of del13q14 and del11q22 was the most frequent complex abnormality (Table 2). 14q32 rearrangements were detected in 14 of 71 patients (20%).

The shortest survival was observed with del11q and del17p and trisomy 12; the longest survival was with del13q14 and in normal patients (p>0.05). The need for medication was significantly higher for del11q22 and del17p (p<0.05). Homozygous del13q14 showed twofold shorter OS (p>0.05) and was categorized in the high-risk group (p<0.05) (Table 3). Positive 14q32 rearrangements showed a twofold increase in mortality and need for medication (p>0.05). They were categorized in the intermediate- to high-risk group (p<0.05).

FISH results were correlated with MRS. The 11q22 and 17p13 deletions had an advanced stage (p<0.05), as well as higher WBC and absolute lymphocyte counts (p<0.05). No difference was observed within groups with respect to b2 microglobulin and LDH and initiation of therapy (p>0.05) (Table 1).

### Results of CLLU1 Expression

**Figure 1:**
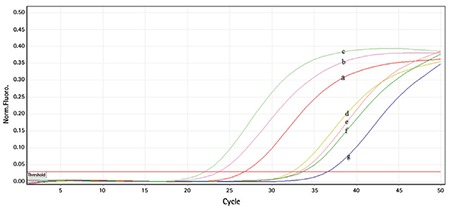
Levels of CLLU1 expression: a, b, d, g- patients; c- standard; e, f- healthy controls.

*CLLU1* expression represented a continuum ranging from 0.1 to 3900 and a median of 17.6-fold upregulation (Figure 1). In the group with high *CLLU1* expression, survival time was twofold lower and the need for medication was twofold higher (p>0.05). High *CLLU1* expression was associated with higher WBC count. There was no correlation between *CLLU1* expression and FISH anomalies, b2 microglobulin and LDH levels, or MRS (p>0.05).

## Discussion

Genetic markers have been major factors in the prognostic evaluation of CLL. The chromosomal anomaly detection rate with FISH is 70%-80% [3]. In our study, the FISH abnormality rate was 62%. Detected abnormalities include del13q14 (40%-60%), trisomy 12 (15%-20%), del11q22 (10%-20%), and del17p13 (5%-10%). Our study yielded a similar pattern. Survival was significantly shorter among patients with del11q12 and del17p13. Similar to the literature data, significant correlation was observed between these two deletions and poor prognosis [5,6,12]. In this study, patients with positive 14q32 rearrangements also had poor outcomes, as shown in some previous reports [13,14].

Few studies refer to homozygote del13q14, and its contribution to prognosis is unclear. Some have reported that homozygote del13q14 is associated with an advanced stage [15,16], while Puiggros et al. [17] noted the opposite. In our study, homozygote del13q14 was correlated with advanced stage and shorter survival.

Previous studies reported that *TP53*, *NOTCH*, *SF3B1*, and *BIRC3* mutations are accountable for poor prognosis [7,8]. The impact of *CLLU1* expression as a new prognostic factor in CLL is unclear. In the present report, high *CLLU1* expression indicated shorter survival and higher need for treatment. Similar results were observed in the literature [11,18,19]. 

In our study, there was no correlation between *CLLU1* expression and FISH aberrations. Some have reported that patients with del17p13 and del11q22 have significantly higher levels of *CLLU1* [11,18]. Chen et al. [20] noted the opposite. Buhl et al. [21] reported no increase in the level of *CLLU1* in patients with trisomy 12; Gonzalez et al. [19] noted the opposite. There was no correlation between trisomy 12 and *CLLU1* expression in our study (Table 4).

## Conclusion

A chromosomal evaluation is still needed for the genetic evaluation of CLL because it can identify unique translocations or aberrations in which breakpoints could lead to identification of new molecular markers. Application of a FISH panel including probes aiming to detect homozygous del13q14, del11q22, del17p, 14q32 rearrangements, and trisomy 12 should still be the routine. The impact of testing *CLLU1* expression is not yet clear and there is a need for more relevant data.

## Figures and Tables

**Table 1:**
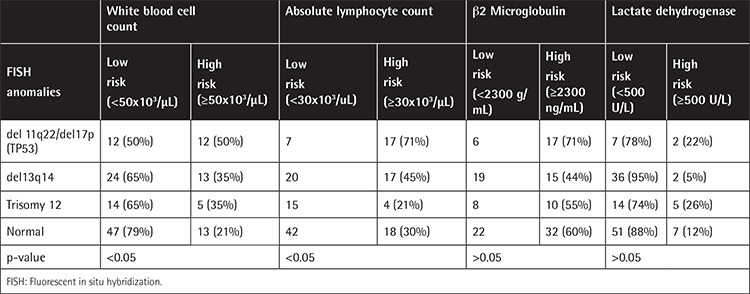
Distribution of patients according to risk groups and chromosomal abnormalities (fluorescent in situ hybridization).

**Table 2:**
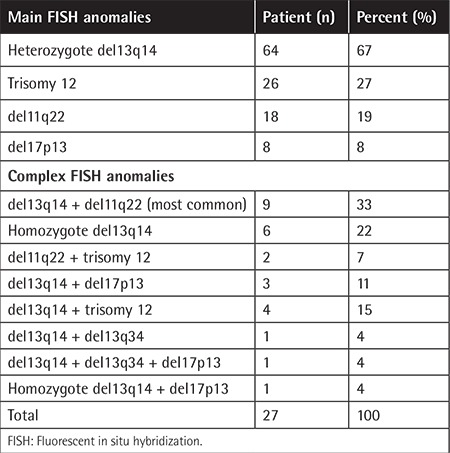
Frequencies of fluorescent in situ hybridization anomalies in chronic lymphocytic leukemia patients.

**Table 3:**
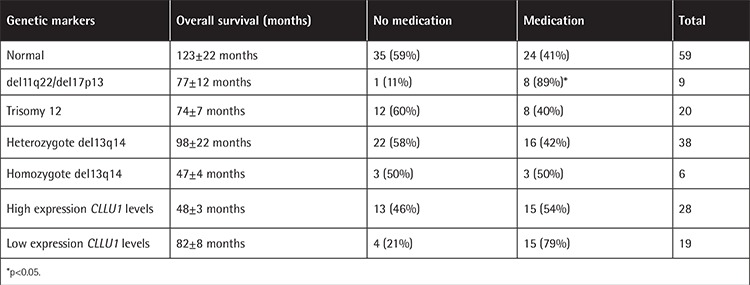
Correlation of the genetic markers with overall survival and medication.

**Table 4:**
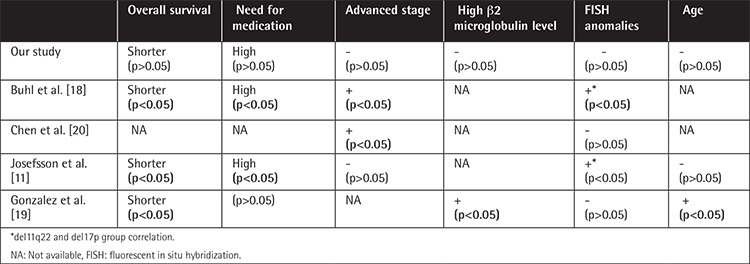
Comparison of prognostic markers in the group with high CLLU1 expression with the findings of previous studies.
